# Income Tax

**Published:** 1913-03

**Authors:** 


### BUFFALO MEDICAL JOURNAL

**A Monthly Review of Medicine and Surgery**

EDITOR AND PUBLISHER DR. A. L. BENEDICT, 228 Summer St., corner of Elmwood Ave., Buffalo. (Address for all communications. Please make personal and telephone calls before 1 P. M.)

THE BUFFALO MEDICAL JOURNAL is the third, in age, in the western continent. It is independent but supports professional organization. As a scientific medium, it is unrestricted in territory. As a news medium, it is limited mainly to the western four districts of the Medical Society of the State of New York. The co-operation of physicians is asked in securing personal, obituary and other news items. Secretaries of Medical Societies in this region are requested to send brief reports of meetings. Full transactions will be published at cost of composition.

Subscription $2.00 a year in advance; $3.00 for two years in advance. Changes and errors in address or failure or duplication of delivery, should be reported immediately, Back numbers will be supplied if possible but requests for copies should be made promptly,

YEARLY VOLUME 68. MARCH, 1913 No. 8

*There is something wrong about the man who could be used either as the literary type or caricature of his profession; and something equally wrong about the man whom no one could conceive as a representative of that profession.*

### Income Tax

Ratifications having been obtained from the requisite number of states, the United States will probably soon enact a law providing for a one per cent. tax on all incomes in excess of $5,000. Unfortunately, this will not affect much more than five per cent. of our profession, but, with the experience of the past in mind, we suggest that the organized profession should take action to secure a fair assessment on net income, exclusive of legitimate business expenses, as for other businesses and professions. In passing, it may not be out of place to suggest also, certain general principles of taxation that ought to be made political issues, irrespective of party.

1. The total tax should not be increased without urgent cause, except in proportion to the increase of population and wealth.

2. A new tax should not supplement but replace, in whole or part, a previous method.

3. No tax should be exorbitant. A tax of 5 cents on a 2 cent Dutch cigar, and then some more for the revenue stamp on the box, is pretty near the old Roman limit of extortion from conquered tribes. A thirty per cent. income tax on real estate for the city alone, plus a six per cent. tax for the county and state, plus transfer taxes occasionally, and then plus one per cent. on the net income if it exceeds the amount that the U. S. Government may fix, is not exactly compatible with the Fourth-of-July oratory about a free, home-owning people.

4. Multiple taxation, either in the sense of taxing two or more persons for the same property, as is uniformly practiced in regard to mortgaged property; or in the sense of taxing the same person several times over on the same property, as illustrated above, should not be allowed.

